# Effects of the Long-Term Consumption of a High-Sucrose Diet on microRNA Expression in Visceral Adipose Tissue of Rats

**DOI:** 10.3390/nu14173465

**Published:** 2022-08-24

**Authors:** Isabela Costa Fernandes, Talita Adriana Pereira Santos, Daiane Teixeira Oliveira, Victor Fernandes Oliveira, Graziele Galdino Sousa, Luciene Santos Pereira, Natália Rocha Barboza, Elísio Alberto Evangelista, Renata Guerra-Sá

**Affiliations:** 1 Instituto de Ciências Exatas e Biológicas (ICEB), Laboratório de Bioquímica e Biologia Molecular (LBBM), Programa de Pós-graduação em Ciências Biológicas, Núcleo de Pesquisa em Ciências Biológicas (NUPEB), Universidade Federal de Ouro Preto (UFOP), Ouro Preto 35400-000, Minas Gerais, Brazil; 2 Instituto de Ciências Exatas e Biológicas (ICEB), Laboratório de Bioquímica e Biologia Molecular (LBBM), Programa de Pós-graduação em Ciências Farmacêuticas, Departamento de Farmácia, Universidade Federal de Ouro Preto (UFOP), Ouro Preto 35400-000, Minas Gerais, Brazil; 3 Faculdade de Ciências Farmacêuticas, Programa de Pós-graduação em Farmácia (Fisiopatologia e Toxicologia), Universidade de São Paulo (USP), São Paulo 05508-000, São Paulo, Brazil

**Keywords:** microRNAs, adipose tissue, rats, sucrose, target prediction, nutritional genomics, high sucrose diet

## Abstract

Noncoding microRNAs are involved in lipid and carbohydrate metabolism pathways and are powerful regulators of gene expression. The goals of this study were to evaluate the temporal expression profiles of miRNAs in rat adipose tissue and predict mRNA–microRNA interactions. Newly weaned Wistar rats were divided into groups fed a standard diet and high-sucrose diet (HSD). The HSD contains 66.86% carbohydrates (40.45% standard diet, 40.45% condensed milk, and 8.58% crystal sugar), and the HSD was provided for 4, 8 and 15-week periods to investigate the expression levels of miRNAs in visceral adipose tissue using RT–qPCR. Target selection, enriched pathways and networks were analyzed in silico. The factor consumption time significantly was associated to decreases (*p* < 0.05) in the expression levels of the following miRNAs: 124-5p, 125-5p, 126-5p, 200c-3p, and 212-3p in all experimental groups. The factor diet significantly influenced rno-miR-124-5p, 200c-3p, and 212-3p expression (*p* < 0.05). A significant reduction (*p* < 0.05) in rno-miR-27a-3p expression was observed. The biological processes involved key pathways regulating fat deposition. Our findings provide important insights into downregulated miRNA expression patterns in visceral adipose tissue, adiposity level, hyperinsulinemia and increased VLDL-c and triglyceride levels.

## 1. Introduction

The World Health Organization (WHO) developed guidance on the consumption of free sugars based on the effect of free sugar intake on weight gain. For adults and children, the intake of free sugars (including monosaccharides and disaccharides added to foods and beverages and sugars that are naturally present in honey, syrups, fruit juices and concentrated fruit juice) is recommended to be reduced to less than 10% of the total energy consumption [1].

MicroRNAs (miRNAs) are 18–22 nucleotide noncoding RNAs that regulate the expression more than 60% of genes involved in almost all physiological and pathological processes [2]. Fat deposition is a complex biological process regulated by multiple factors, including miRNAs [3]. Previous studies have described the effects of a high-sucrose diet (HSD) on lipid metabolism. This research is justified because the consumption of an HSD increased both adipocyte size and number in rat retroperitoneal adipose tissue by simultaneously upregulating pro-adipogenic signals (the PPARγ pathway) and downregulating anti-adipogenic signals (Wnt pathway) in young rats [4].

To date, many studies have shown that miRNAs are important regulators of proliferation, adipogenic determination and differentiation, apoptosis and metabolism in adipocytes [3]. For example, miR-125a-5p promotes proliferation and inhibits adipogenic differentiation by negatively regulating STAT-3 (signal transducer and activator of transcription-3) in the 3T3-L1 preadipocyte cell line [5]. In the present study, in vivo experiments were performed to determine whether HSD affects miRNA expression in visceral adipose tissue. We also analyzed the effect of the HSD consumption time by comparing the expression profiles between rats with four, eight and fifteen weeks of HSD consumption. In addition, we also used computational approaches to identify the interaction between miRNAs and their mRNA targets and then predicted the effects of up- or downregulation of miRNA expression induced by HSD compared with a balanced diet.

## 2. Materials and Methods

### 2.1. Animals and Diet

All experimental procedures performed in the present study were approved by the Committee on Ethics in the Use of Animals (CEUA) under protocol 2016/26. Newly weaned Wistar rats (twenty-one days) from the Animal Science Center were divided into six groups: standard diet (STD), *n* = twenty; and high sucrose diet (HSD), *n* = twenty-four. One group received a standard diet for defined experimental periods: four weeks (*n* = six), eight weeks (*n* = six) and fifteen (*n* = eight) weeks. The other group received a high-sucrose diet for the same defined experimental periods: four (*n* = six), eight (*n* = six) and fifteen (*n* = twelve) weeks.

The standard Nuvilab diet (STD) that was used in the study consisted of 57.15% carbohydrates, 28.09% proteins and 14.75% lipids (Table 1). The high-sucrose diet (HSD) is considered “palatable” because it contains condensed milk, and it consists of 40.45% standard diet, 40.45% Nestlé condensed milk, 8.6% crystal sugar and 10.5% water, with 66.86% carbohydrates, 17.89% proteins and 15.26% lipids. The total calorie calculation for both diets considered 4 kcal/1 g of carbohydrate, 4 kcal/1 g of protein and 9 kcal for each 1 g of lipid. The percentage of macronutrients considered calories provided by each macronutrient in relation to the caloric total for every 100 g of prepared HSD contained 286.36 kcal, and every 100 g of STD diet contained 308.52 kcal (Table 1). During all experimental periods, both diets and water were provided ad libitum, and the diet was weighed and replenished after consumption. The light/dark cycle was maintained for twelve hours each. The body mass of each animal evaluated at the beginning and end of the experiment.

After a twelve hour fast, blood samples were collected on the day of sacrifice, and visceral, inguinal, epididymal and brown adipose tissue samples were collected, weighed, frozen in liquid nitrogen and stored at −80 °C. The adiposity index was calculated as the sum of mass adipose fat pads in grams divided by body weight in grams × 100.

### 2.2. Biochemical Parameters

Total cholesterol, HDL-c, LDL-c, VLDL-c, triglyceride (TG), fasting glucose and insulin levels were evaluated. The respective BIOCLIN enzyme kits (Bioclin/Quibasa) were used according to the manufacturer’s protocol, and the measurements were recorded using spectrophotometry with an automated Random Access Clinical Analyzer (model Wiener Lab, CM 200, São Paulo, Brazil). The serum insulin concentration was determined using a Rat/Mouse Insulin ELISA Kit (Millipore, St. Charles, MO, USA, Cat. # EZRMI-13K), according to the manufacturer’s recommendation.

### 2.3. Analysis of Gene Expression

#### 2.3.1. MicroRNA Extraction from Visceral White Adipose Tissue

Frozen visceral adipose tissue (170 mg) was weighed, and total RNA enriched in microRNAs was extracted using the miRNeasy (Qiagen, Hilden, Germany) kit according to the manufacturer’s instructions. After determining the RNA concentration using a Nanodrop spectrophotometer, the quality of the extracted RNA was evaluated by separation on a 2% agarose gel.

#### 2.3.2. cDNA Synthesis

The first cDNA strand was synthesized from 1 µg of total RNA enriched in microRNAs extracted from visceral adipose tissue using the miScript II Reverse Transcription Kit (Qiagen, Hilden, Germany), according to the manufacturer’s recommendations.

#### 2.3.3. Quantitative Real-Time Polymerase Chain Reaction (RT–qPCR)

The analysis of microRNA expression was performed using RT–qPCR, according to MIQE. The reactions were performed using the SYBR Green detection Kit in 96-well plates sealed with optical adhesive at the end of the procedure. Two microliters of primers (at 2.5 μM concentration), 2 μL of cDNAs diluted 10-fold, 1 μL of 10× miScript Universal Primers and 5 μL of 2× QuantiTect SYBR Green PCR Master Mix were pipetted in a 10 μL reaction in each well. All assays were performed in biological triplicates for the evaluated microRNAs, and the endogenous control, the spliceosome small nuclear RNA U6, was present in all plates. For each miRNA examined at each time point, delta Ct (ΔCt) was calculated as (CtU6—CtmiRNA) and reported in copy numbers. RT–qPCR was performed using an ABI 7300 instrument from Applied Biosystems. Based on studies examining adipogenic processes, rno-mir-27a-3p, rno-mir-124-5p, rno-mir-125-5p, rno-mir-126-3p, rno-mir-126-5p, rno-mir-200c-3p, rno-mir-212-3p, and rno-mir-221-3p were identified (Table 2).

### 2.4. Target Selection, Network and Enriched Pathways

The predicted targets were obtained for the eight microRNAs using the miRDataBase (miRDB) tool. A total of 2710 targets were identified, and the first 45 scores for seven microRNAs were selected for this study. In addition, microRNA 126a-3p was found and only had 5 targets in the database. Three hundred and twenty targets were selected. Gephi Software was used to generate the network of the eight microRNAs and their 320 selected targets. The enrichment analysis of the main biological processes related to the selected targets was performed with the Funset software tool. Significantly, for biological processes, the *p* value < 0.05 was corrected with the FDR for multiple tests, and it was tabulated by the enrichment score. The analysis of the main molecular function related to the selected targets was performed with the Pather algorithm in Network Analyst software.

### 2.5. Statistical Analysis

The analysis was performed using GraphPad Prism 8 software. The normality of all data was verified with the Kolmogorov–Smirnov test. The differences between groups were evaluated with two-way ANOVA followed by Bonferroni’s post hoc test to compare the means of the biochemical parameters and the relative expression of microRNAs, with the type of diet and number of weeks (time) serving as the factors.

## 3. Results

### 3.1. Nutritional Effects of a High-Sucrose Diet on Variations in Body Mass Gain, Adiposity Index and Biochemical Parameters in Wistar Rats during Development

**Figure 1:**
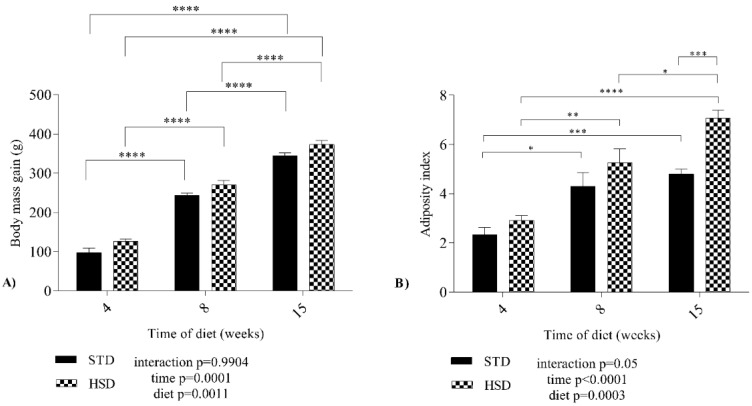
**Effect of a high-sucrose diet on the variations in body mass gain and adiposity index of Wistar rats.** (**A**) Body mass gain and (**B**) adiposity index for both groups between the three periods of diet consumption. STD: standard diet; HSD: high-sucrose diet. Means were compared using two-way ANOVA with Bonferroni’s post hoc test. *: *p* ≤ 0.05; **: *p* ≤ 0.01; ***: *p* ≤ 0.001; ****: *p* ≤ 0.0001.

Data related to the growth of the experimental groups are presented in Figure 1A, which shows the body mass gain during three periods (four, eight and fifteen weeks). The time (*p* = 0.0001) and the diet (*p* = 0.0011) factors affected body mass gain. No significant interaction (*p* = 0.99) was observed between time and diet; however, the body mass gain increased by a mean value of 146 g from four weeks to eight weeks in the HSD group (*p* < 0.05), and by 101 g from eight weeks to fifteen weeks in the HSD group (*p* < 0.05).

Figure 1B presents the adiposity index of rats fed both diets during development. The HSD increased the mean adiposity index of rats of the same age (*p* < 0.001). During the development of the STD group, the mean adiposity index increased significantly (*p* < 0.05) from 4 to 8 weeks and from 4 to 15 weeks. In the HSD group, an increase (*p* < 0.05) in the mean adiposity index was also observed between the three stages, with the highest mean value recorded at 15 weeks. A significant effect of the interaction (*p* = 0.05) between time and diet on the adiposity index was not observed.

Our results reinforce the findings reported in the literature about body mass gain and adiposity index. We showed that while weight gain occurred over the experimental period and the adiposity index increased at 4 and 8 weeks, regardless of the type of diet, the adiposity index increased at 15 weeks due to the consumption of the HSD.

Table 3 shows the effects on the biochemical parameters after the consumption of a high-sucrose diet for three different periods.

A significant effect of the interaction (*p* < 0.05) between diet and time on HDL-c, VLDL-c and TG levels was observed. The time factor significantly altered (*p* < 0.05) insulin, total cholesterol, HDL-c, VLDL-c and TG levels. The diet factor significantly (*p* < 0.05) altered HDL-c, LDL-c and total cholesterol, triglyceride (TG) and VLDL-c levels.

Fasting glucose levels were modulated by the time factor in the STD group and exhibited a mean reduction of 2.2 mmol/L (*p* < 0.05) from 4 to 8 weeks and a mean reduction of 2.9 mmol/L from 4 to 8 weeks. However, fasting glucose levels did not change in rats fed the HSD over time (*p* > 0.05). Fasting insulin levels showed an increase of 2.006 ng/mL (*p* < 0.05) in the HSD group at 8 weeks of the experiment when compared with 4 weeks in the STD group, suggesting that rats fed the HSD for 8 weeks had hyperinsulinemia.

A significant effect of the interaction (*p* < 0.05) of diet and time factors on the levels of HDL-c, VLDL-c and TG was observed, with higher HDL-c levels (*p* < 0.05) detected after 15 weeks of STD feeding and higher serum levels of VLDL-c (*p* < 0.05) and TG detected at 15 weeks of HSD feeding (*p* < 0.05).

When comparing groups fed the HSD at different time points, the VLDL-c level had an increase of 10.32 mg/dL between 4 and 15 weeks of HSD consumption (*p* < 0.05), had an increase of 13.32 mg/dL (*p* < 0.05) between 8 and 15 weeks of HSD consumption, and had an increase of 12.72 mg/dL (*p* < 0.05) between 15 weeks of STD and 15 weeks of HSD consumption.

Between 4 and 15 weeks of HSD consumption, the triglyceride levels had an increase of 50.70 mg/dL (*p* < 0.05). Additionally, triglyceride levels had an increase of 66.70 mg/dL from 8 to 15 weeks of HSD consumption (*p* < 0.05) and had an increase of 64.10 mg/dL in the group fed the HSD for 15 weeks compared to the group fed the STD for 15 weeks (*p* < 0.05).

The STD increased the mean serum levels of HDL-c by 9 mg/dL (*p* < 0.05) from 4 to 15 weeks. The HSD reduced the mean serum levels of HDL-c in all periods (4 weeks of HSD versus 15 weeks of STD-—reduction of 11 mg/dL (*p* < 0.05), 8 weeks of HSD versus 15 weeks of STD-—reduction of 14 mg/dL (*p* < 0.05), and in the comparison between 15 weeks of STD versus 15 weeks of HSD, the reduction was 10 mg/dL (*p* < 0.05).

In addition, the diet influenced LDL-c levels; in the comparison between the 15-week STD and 15-week HSD groups, the reduction was 30.82 mg/dL (*p* < 0.05). No interaction between the factors was observed for LDL-c levels (*p* > 0.05), but the isolated factors diet (*p* < 0.001) and time (*p* < 0.001) contributed to changes in LDL-c levels.

No effect of interaction between the factors on total cholesterol levels was observed (*p* > 0.05), but the isolated factors diet (*p* = 0.01) and time (*p* < 0.0001) contributed to total cholesterol levels. In the comparison of the 15-week STD and 4-week HSD groups, a reduction of 34.80 mg/dL in total cholesterol levels was observed (*p* < 0.05). A reduction of 46.8 mg/dL (*p* < 0.05) in total cholesterol levels was observed in the comparison of the 15-week STD group and 8-week HSD group, and the reduction in total cholesterol levels was 35.21 mg/dL (*p* < 0.05) in the comparison of the 15-week STD group with the 15-week HSD group. However, no change in total cholesterol levels was observed between the HSD groups compared at the three periods (*p* > 0.05).

### 3.2. Time-Dependent Effects of High-Sucrose Diet Consumption on microRNA Expression

**Figure 2:**
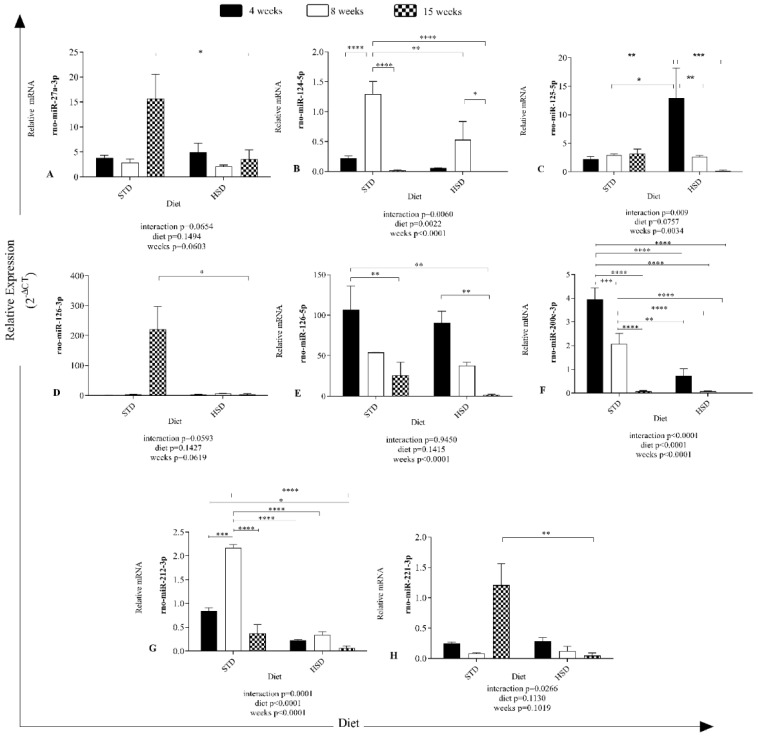
**Effects of the time of consumption of a high-sucrose diet on the expression of microRNAs in visceral white adipose tissue**. Differences in the abundances of microRNAs in both groups between the three periods of diet consumption. (**A**) Relative rno-miR-27a-3p expression (2^−ΔCT^); (**B**) relative rno-miR-124-5p expression (2^−ΔCT^); (**C**) relative rno-miR-125-5p expression (2^−ΔCT^); (**D**) relative rno-miR-126-3p expression (2^−ΔCT^); (**E**) relative rno-miR-126-5p expression (2^−ΔCT^); (**F**) relative rno-miR-200c-3p expression (2^−ΔCT^); (**G**) relative rno-miR-212-3p expression (2^−ΔCT^); (**H**) relative rno-miR-221-3p expression (2^−ΔCT^). STD—standard diet; HSD—high-sucrose diet; rno—*Rattus norvegicus*; miR—microRNA. Means were compared using two-way ANOVA with Bonferroni’s post hoc test. *: *p* ≤ 0.05; **: *p* ≤ 0.01; ***: *p* ≤ 0.001; ****: *p* ≤ 0.0001.

The results in Figure 2 show different expression profiles of microRNAs in visceral white adipose tissue of rats after the consumption of a high-sucrose diet during development.

An effect of the interaction between the type of diet and time of diet consumption on rno-miR-124-5p (*p* = 0.006), rno-miR-125-5p (*p* = 0.009), rno-miR-200c-3p (*p* < 0.0001), rno-miR-212-3p (*p* = 0.0001) and rno-miR-221-3p (*p* = 0.02) expression was observed (Figure 2).

The consumption time significantly decreased rno-miR-124-5p (*p* < 0.0001), 125-5p (*p* = 0.0034), 126-5p (*p* < 0.0001), 200c-3p (*p* < 0.0001), and 212-3p (*p* < 0.0001) expression in the six groups (Figure 2).

Significant effects of the diet were verified on rno-miR-124-5p (*p* = 0.0022), 200c-3p (*p* < 0.0001), and 212-3p (*p* < 0.0001) expression (Figure 2).

In addition, a significant reduction of 12 copies of rno-miR-27a-3p expression was found (Figure 2A) in rats with 15 weeks of HSD feeding compared with the STD group along with a reduction of 12 copies of rno-miR-27a-3p expression (*p* < 0.05).

The STD increased the mean expression of rno-miR-124-5p by 1.077 copies (*p* < 0.0001) (Figure 2B) from 4 to 8 weeks and decreased the value by 1.276 from 8 to 15 weeks (*p* < 0.0001). The HSD reduced the mean rno-miR-124-5p expression (8 weeks of STD versus 4 weeks of HSD-—reduction of 1.241 copies (*p* < 0.0001), 8 weeks of STD versus 8 weeks of HSD-—reduction of 0.7667 copies (*p* < 0.05), and 8 weeks of HSD versus 15 weeks of HSD-—reduction of 0.5293 copies (*p* < 0.05).

The HSD increased the mean number of rno-miR-125-5p copies by 10.72 (*p* < 0.05) (Figure 2C) after 4 weeks of consumption compared with the STD group, decreased the copy number by 10.31 from 4 to 8 weeks in the HSD group (*p* < 0.05) and decreased the copy number by 12.76 from 4 to 15 weeks in the HSD group (*p* < 0.05).

The HSD decreased the mean copy number of rno-miR-126-3p (15 weeks of STD versus 15 weeks of HSD-reduction of 217.2 copy numbers (*p* < 0.05)). Notably, rno-miR-126-3p (Figure 2D) was the microRNA with more copies in the STD group at 15 weeks and was overexpressed; however, rno-miR-126-5p expression was the second highest overexpressed miRNA at all time points.

No effect of Ihe interaction (*p* > 0.05) between the factors (diet and time) was observed on rno-miR-126-5p expression (Figure 2E), and diet (*p* > 0.05) did not alter the abundance of microRNAs, but the factor time (*p* < 0.0001) contributed. The expression of rno-miR-126-5p was reduced by 81.11 copy numbers between 4 and 15 weeks in the STD group (*p* < 0.05), by 105.2 copy numbers (*p* < 0.05) between the STD group at 4 weeks and the HSD group at 15 weeks, and by 88.45 copy numbers between 4 and 15 weeks in the HSD group (*p* < 0.05).

The expression of rno-miR-200c-3p was reduced by the interaction (*p* < 0.0001) between the factors (diet and time) (Figure 2F) and the isolated factors diet (*p* < 0.0001) and time (*p* < 0.0001). Its expression exhibited a reduction of 1.884 copies between 4 and 8 weeks in the STD group (*p* < 0.05), 3.877 copies between 4 and 15 weeks in the STD group (*p* < 0.0001), and 1.993 (*p* < 0.0001) copies between 8 and 15 weeks in the STD group. In addition, a reduction of 3.232 copies was observed at 4 weeks between the STD and HSD groups (*p* < 0.0001) and of 1.994 copies at 8 weeks between the STD and HSD groups (*p* < 0.0001).

In the comparison of rno-miR-212-3p (Figure 2G) expression, an increase of 1.328 copies was observed between 4 and 8 weeks in the STD group (*p* < 0.0001), 1.795 copies between 8 and 15 weeks in the STD group (*p* < 0.0001), and 1.827 (*p* < 0.0001) copies at 8 weeks between the STD and HSD groups.

The HSD decreased the mean rno-miR-221-3p expression (15 weeks of STD versus 15 weeks of HSD-reduction of 1.162 copies (*p* < 0.05), as shown in Figure 2H.

### 3.3. microRNA–mRNA Interactions and Regulatory Network Predicted in Rats with Long-Term Consumption of a High-Sucrose Diet

**Figure 3:**
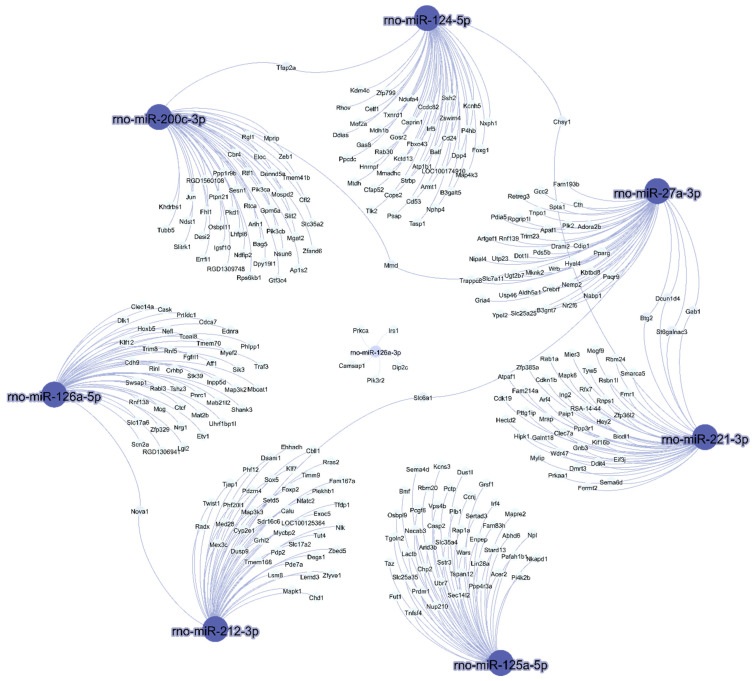
**In silico prediction of microRNA targets and microRNA–mRNA interaction networks.** The interactions between eight miRNAs and their predicted mRNA targets were rendered using Gephi.

The interaction between predicted mRNA targets and eight miRNAs expressed in visceral white adipose tissue of rats after the consumption of a high-sucrose diet is shown in Figure 3. More than one microRNA was identified for the same target gene.

Notably, rno-miR-27a-3p was the miRNA with the greater number of interactions (Figure 3); it shares a common target with rno-miR124-5p, namely, *Chsy1*, and shares a second common target with rno-miR-200c-3p, namely, *Mmd*. Moreover, it shares the targets *Dcun1d4*, *Gab1*, *Btg* and *St6galnac3* with rno-miR-221-3p, and the common target *Slc6a1* with rno-miR-212-3p. Additionally, rno-miR-212-3p shares a common target, *Nova1*, with rno-miR-126-5p. Finally, rno-miR-124-3p interacted with rno-miR-200c-3p by targeting *Tfap2a*.

**Figure 4:**
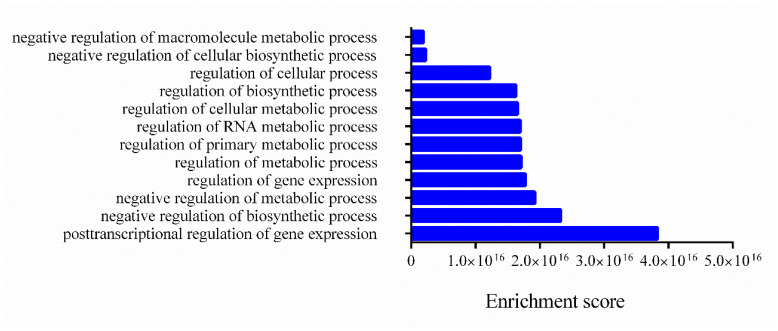
**Biological processes related to mRNA targets.** The enrichment scores of biological processes annotated and related to mRNA targets were evaluated using Funset.

**Figure 5:**
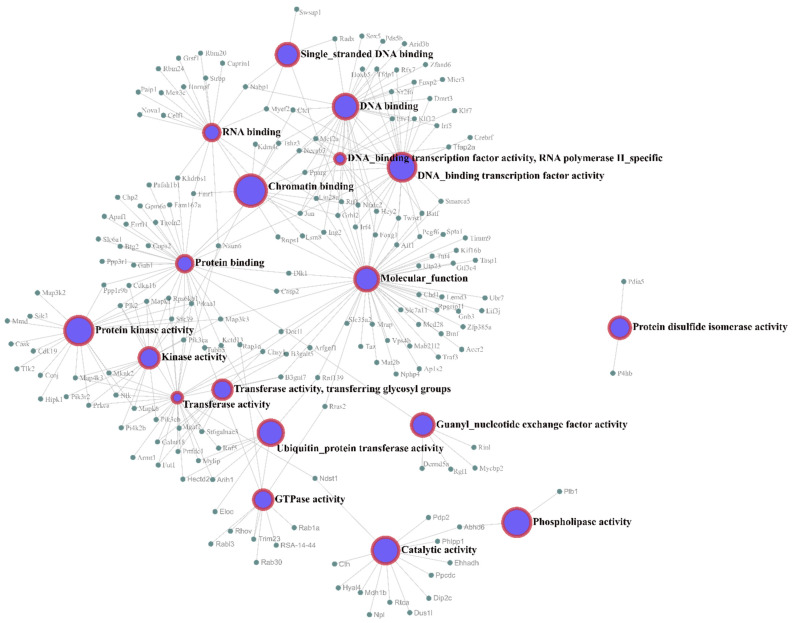
**Molecular function network of the mRNA targets.** The interaction network between the mRNA targets and their molecular functions was rendered using Network Analyst software.

Gene Ontology (GO) enrichment analyses of the target genes were performed for two different aspects, namely, biological processes (Figure 4) and molecular functions (Figure 5), and the most highly enriched GO categories were plotted. More than one molecular function was identified for the same target gene.

In Figure 4, the clusters of biological processes are presented, and most annotations are related to post-transcriptional regulation of gene expression and negative regulation of biosynthetic processes and metabolic processes.

Figure 5 shows the molecular function network of the target genes. Most of the molecular function annotations of the targets are related to transferase activity, protein binding, and DNA binding.

## 4. Discussion

Experiments have revealed that microRNAs may have important roles and functions in adipocyte biology [6,7,8]. However, the temporal effect of HSD consumption on miRNA expression in rat visceral adipose tissue is still poorly understood. Protein products of the mRNA targets of microRNAs and their interactions may help to determine the protein landscape of obesity, and modulators, key pathways, interactomes and novel therapeutic targets must be identified because obesity involves a complex signaling network.

According to previous studies, fat accumulation occurs in the abdominal region of adult male rats from 90 days or 12 weeks of age [9]. Here, the growth was normal in both groups and adiposity increased with age.

In relation to biochemical parameters, serum total cholesterol, LDL-c and HDL-c levels in the HSD group decreased at all time points because the diet contains a lower percentage of fat. The propensity to increase insulin secretion, without altering fasting glucose, direct energy storage as fat in adipose tissue, and inhibition of lipid oxidation by metabolically active tissues reflects the processes we suggest to occur in our model. Additionally, in the present study, we observed significant increases in serum triglyceride and VLDL-c levels, which indicates that the small increase in the amount of added sugar in the HSD group altered the lipolysis/lipogenesis balance.

Advances in bioinformatic tools can help more easily to identify nutritional gaps. The expression pattern of dysregulated miRNAs is not always constant with time, and similar fluctuations were reported in the literature [10]. Age regulates the expression of microRNAs, and this fluctuation in expression levels may be important to maintain low adiposity, as verified for the 8-week STD. Younger rats fed the STD displayed a higher abundance of microRNAs, which may be related to low adiposity and hyperinsulinemia.

The differential expression of 26 miRNAs was observed compared to adipose tissue of animals treated with control diets. Among the evaluated microRNAs, the mmu-miR200 family, mir200a, mir 200b, and mir200c were downregulated [11], which reinforces the low copy number of rno-miR-200c-3p expression in the HSD group in the present study.

Evidence suggests that diets may influence the risk of disease development by modulating miRNA expression. A high-fat diet is known to induce differential expression [11], and the present study showed that a high-sucrose diet also induces adiposity and alters the expression of key miRNAs in obese animals.

According to Table 1, the standard diet has a higher fiber content because it contains FODMAPs (Fermentable Oligo-, Di-, Monosaccharides, Polyols-abbreviations for Oligosaccharides, Disaccharides, Monosaccharides and Fermentable Polyols), which are fermentable carbohydrates that are present in a large amount of food and are even present in a reduced amount in a high-sucrose diet. A standard diet contains more corn, soy, and wheat, which are carbohydrates that are not easily absorbed by the small intestine and are fermentable, producing gases.

Studies have indicated that the increase in visceral adipose tissue may be associated with low consumption of polysaccharides (prebiotics), which, when fermented, would induce the constitution of the beneficial microbiota composed of probiotic bacteria. The consumption of a diet rich in sucrose for 15 weeks induced the enrichment of bacteria of the phylum Proteobacteria, which are associated with the development of obesity [12].

More than one microRNA targeted for the same gene in the network was shown. The target *Chsy1*, which encodes a hexosyltransferase, catalyzes the reaction: D-glucuronyl-N-acetyl-1,3-beta-D-galactosaminylproteoglycan + UDP-N-acetylgalactosamine = N-acetyl-D-galactosaminyl-1,4-beta-D-glucuronyl-N-acetyl-1,3-beta-D-galactosaminylproteoglycan + UDP. The target *Mmd* encodes a monocyte to macrophage differentiation factor that may be activated in response to inflammation mediated by macrophages.

The post-transcriptional regulation of gene expression is considered any process that modulates the frequency, rate or extent of gene expression after the production of an RNA transcript, suggesting that a high frequency of RNA transcripts was not inhibited by microRNA downregulation. The regulation of cellular metabolic process is defined by any process that modulates the frequency, rate or extent of the chemical reactions and pathways by which individual cells transform chemical substances, such as the inhibition of fatty acid beta-oxidation in adipocytes using acyl-CoA dehydrogenase, a sublevel of the regulation of the cellular metabolic process ontology, which reinforces the increased VLDL-c and triglyceride levels.

The expression of mRNA targets is upregulated and is suggest because of the downregulation of the eight miRNAs evaluated. The molecular functions protein binding and DNA binding were identified as related to these target genes.

Transferase activity, shown in the network, can be, here, related to the conversion of pyruvate to acetyl-CoA, which involves the transfer of a CoA group, in which the reaction is upregulated, as well as examples of transferase activity and the addition of methyl and glycosyl groups, which corroborate the increased triglyceride levels in the HSD group. In the present study, the molecular functions protein binding and DNA binding are important for the gene silencing mechanism of microRNAs, suggesting the overexpression of mRNA targets related to glucose and lipid metabolism.

More experiments should be performed to determine the protein levels and circulating miRNA expression levels. We hope that future studies will focus on dimorphic miRNA expression patterns and correlate these patterns with obesity, and these results can be used as the basis for new experiments evaluating serum microRNAs and exosomic properties.

## 5. Conclusions

In conclusions, we provide deeper insights into the time-dependent variation in biochemical parameters and rno-miR-27a-3p, rno-miR-124-5p, rno-miR-125a-5p, rno-miR-126a-3p, rno-miR-126a-5p, rno-miR-200c-3p, rno-miR-212-3p and rno-miR-221-3p expression in adipose tissue associated with the long-term consumption of a high-sucrose diet. Our findings provide important insights into downregulated miRNA expression patterns in visceral adipose tissue, adiposity level, hyperinsulinemia and increased VLDL-c and triglyceride levels.

## Figures and Tables

**Table 1:** Centesimal composition of diets.

Centesimal Composition		
	STD	HSD
Energy (kcal/g)	3.08	2.86
Nutrients (g/100g)		
Carbohydrate	44.08	47.86
Protein	21.67	12.81
Lipid	5.06	4.85
Water	10.89	25.77
Fiber	11.43	5.08
Mineral	6.87	3.63
Total	100	100
Calorie Percentage (%)		
as Carbohydrate	57.16	66.86
as Protein	28.09	17.89
as Lipid	14.75	15.26
Total	100	100

The centesimal composition can be found in each diet.

**Table 2:** Oligonucleotide sequences according miRDB.

Noncoding RNA	Sequence
snoRNAU6	AGTTGAGGTC ACACGCTGGT CGATGAACTC CTAAGTGTAG GTAGTGTGCT AAACGAGCGG CAAG
rno-miR-27a-3p	UUCACAGUGGCUAAGUUCCGC
rno-miR-124-5p	CGUGUUCACAGCGGACCUUGAU
rno-miR-125a-5p	UCCCUGAGACCCUUUAACCUGUGA
rno-miR-126a-3p	UCGUACCGUGAGUAAUAAUGCG
rno-miR-126a-5p	CAUUAUUACUUUUGGUACGCG
rno-miR-200c-3p	UAAUACUGCCGGGUAAUGAUG
rno-miR-212-3p	UAACAGUCUCCAGUCACGGCCA
rno-miR-221-3p	AGCUACAUUGUCUGCUGGGUUUC

**Table 3:** Effects of a high-sucrose diet on biochemical parameters in male Wistar rats.

Parameters	Unit	Time (Weeks)	STD	HSD	*p* Value		
			M ± SD	M ± SD	Time Effect	Diet Effect	Interaction
Total cholesterol	mg/dL				0.0152	<0.001	0.1462
		4	109 ± 16 ^ab^	96 ± 13 ^b^			
		8	109 ± 26 ^ab^	84 ± 17 ^b^			
		15	130.80 ± 11.31 ^a^	95.60 ± 3.36 ^b^			
LDL-c	mg/dL	4	47 ± 15 ^ab^	31 ± 17 ^ab^	<0.001	<0.001	0.5855
		8	49 ± 18 ^ab^	26 ± 10 ^ab^			
		15	51.87 ± 32.65 ^a^	21.05 ± 9.71 ^b^			
HDL-c	mg/dL	4	43 ± 6 ^a^	41 ± 4 ^a^	0.9377	0.0003	0.026
		8	38 ± 8 ^a^	38 ± 5 ^a^			
		15	52.05 ± 4.8 ^b^	42.02 ± 2.30 ^a^			
VLDL-c	mg/dL	4	17 ± 5.0 ^a^	24 ± 7.0 ^a^	0.0001	<0.0001	0.0036
		8	21 ± 5 ^a^	21 ± 5 ^a^			
		15	21.61 ± 1.7 ^a^	34.32 ± 3.91 ^b^			
Triglycerides	mg/dL	4	86 ± 17 ^a^	119 ± 35 ^a^	0.0001	<0.0001	0.0012
		8	106 ± 23 ^a^	103 ± 28 ^a^			
		15	105.6 ± 8.9 ^a^	169.7 ± 17.55^b^			
Triglyceride/HDL ratio		4	1.82 ± 2.82 ^a^	3.02 ± 7.98 ^a^	0.94	0.90	0.79
		8	2.75 ± 2.8 ^a^	2.71 ± 5.3 ^a^			
		15	4.11 ± 3.94 ^a^	2.28 ± 8.67 ^a^			
Fasting glucose	mmol/L	4	8.7 ± 1.7 ^a^	7.6 ± 2.20 ^ad^	0.0001	0.847	0.0643
		8	6.5 ± 0.9 ^bd^	7 ± 0.20 ^ad^			
		15	5.8 ± 0.2 ^cd^	6.60 ± 0.50 ^d^			
Fasting insulin	ng/mL	4	0.64 ± 0.60 ^a^	1.63 ± 1.42 ^ab^			
		8	1.08 ± 0.80 ^ab^	2.64 ± 2.46 ^b^	0.0937	0.0063	0.4738
		15	0.7 ± 0.32 ^a^	1.2 ± 0.62 ^ab^			
*n*			STD-4w (*n* = 6)	HSD-4w (*n* = 6)			
			STD-8w (*n* = 6)	HSD-4w (*n* = 6)			
			STD-15w (*n* = 8)	HSD-15w (*n* = 12)			

Biochemical parameters were measured in both groups between the three periods of diet consumption. STD: standard diet; HSD: high-sucrose diet. Means (M) were compared using two-way ANOVA with Bonferroni’s post hoc test. Different letters indicate significant differences between the six groups (*p* < 0.05). The same letters indicate no significant difference (*p* > 0.05). a is different to b. SD indicates standard deviation. *n* indicates the sample size by group (time in weeks) and diet.

## Abbreviations

miRNAs—microRNAs, HSD—high sucrose diet and STD—standard diet.
